# Crystal structure of (2,11-di­aza­[3.3](2,6)pyridino­phane-*κ* ^4^ *N*,*N*′,*N*′′,*N*′′′)(1,6,7,12-tetra­aza­perylene-*κ* ^2^ *N* ^1^,*N* ^12^)ruthenium(II) bis­(hexa­fluorido­phosphate) aceto­nitrile 1.422-solvate

**DOI:** 10.1107/S1600536814021060

**Published:** 2014-09-30

**Authors:** Thomas Brietzke, Falko Otto Rottke, Alexandra Kelling, Uwe Schilde, Hans-Jürgen Holdt

**Affiliations:** a Universität Potsdam, Institut für Chemie, Anorganische Chemie, Karl-Liebknecht-Str. 24-25, D-14476 Potsdam, Germany

**Keywords:** crystal structure, coordination compound, ruthenium, stacking

## Abstract

In the title compound, [Ru(C_14_H_16_N_4_)(C_16_H_8_N_4_)](PF_6_)_2_·1.422CH_3_CN, discrete dimers of complex cations, [Ru(L–N_4_H_2_)tape]^2+^ are formed {L–N_4_H_2_ = 2,11-di­aza­[3.3](2,6)pyridino­phane; tape = 1,6,7,12-tetra­aza­perylene}, held together by π–π stacking inter­actions *via* the tape ligand moieties with a centroid–centroid distance of 3.49 (2) Å, assisted by hydrogen bonds between the non-coordinating tape ligand α,α′-di­imine unit and the amine proton of a 2,11-di­aza­[3.3](2,6)-pyridino­phane ligand of the opposite complex cation. The combination of these inter­actions leads to an unusual nearly face-to-face π–π stacking mode. Additional weak C—H⋯N, C—H⋯F, N—H⋯F and P—F⋯π-ring (tape, py) (with F⋯centroid distances of 2.925–3.984 Å) inter­actions are found, leading to a three-dimensional architecture. The Ru^II^ atom is coordinated in a distorted octa­hedral geometry, particularly manifested by the N_amine_—Ru—N_amine_ angle of 153.79 (10)°. The counter-charge is provided by two hexa­fluorido­phosphate anions and the asymmetric unit is completed by aceto­nitrile solvent mol­ecules of crystallization. Disorder was observed for both the hexa­fluorido­phosphate anions as well as the aceto­nitrile solvate mol­ecules, with occupancies for the major moieties of 0.801 (6) for one of the PF_6_ anions, and a shared occupancy of 0.9215 (17) for the second PF_6_ anion and a partially occupied aceto­nitrile mol­ecule. A second CH_3_CN mol­ecule is fully occupied, but 1:1 disordered across a crystallographic inversion center.

## Abstract 2

The highlighted ruthenium complex forms discrete dimers by π–π stacking inter­actions and hydrogen bonds. This combination of inter­actions results in an unusual nearly face-to-face π–π stacking mode.

## Chemical context   

Heteroaromatic ligands with more than three fused rings are commonly called large-surface ligands. Such ligands have attracted attention due to their use as connecting building blocks for supra­molecular assemblies. If large-surface ligands feature more than one ligand donor site, connection between neighboring complexes can be realized through normal metal coordination (Ishow *et al.*, 1998 ▶), but the large π system also allows for strong π–π stacking inter­actions. (Kammer *et al.*, 2006 ▶; Gut *et al.*, 2002 ▶). In order to study the properties of ruthenium complexes containing large-surface ligands, we have recently reported an easy entry to such complexes (Brietzke, Mickler, Kelling, Schilde *et al.*, 2012 ▶). Therein, we formulated the advantages of the 2,11-dimethyl-2,11-di­aza­[3.3](2,6)-pyridino­phane (L–N_4_Me_2_) macrocycle over bi­pyridine (bpy)-type ligands in saturating the coordination sphere of an octa­hedral ruthenium complex containing the large-surface ligand of inter­est. The microwave-assisted synthesis of the precursor [Ru(L–N_4_Me_2_)]^2+^, starting from [Ru(DMSO)_4_Cl_2_] and L–N_4_Me_2_, in an ethano­lic solution finished within 30 min. It is not only fast, but also reproducible with only few byproducts, and hence requires no labor-intensive workup. Moreover, using the *C*_2v_ symmetric macrocycle rather than bi­pyridine-type ligands avoids the formation of mono- and dinuclear complexes with multiple stereoisomeric forms (Brietzke, Mickler, Kelling & Holdt, 2012 ▶; Brietzke *et al.*, 2014 ▶). To test the applicability of our microwave-assisted synthetic strategy for use with other related macrocyclic ligands, we choose the unmethyl­ated parent compound of L–N_4_Me_2_, 2,11-di­aza­[3.3](2,6)-pyridino­phane (L–N_4_H_2_) as a new ligand for Ru^II^. Herein, we present the structure of the complex [Ru(L–N_4_H_2_)tape](PF_6_)_2_, (tape = 1,6,7,12-tetra­aza­perylene), obtained as its aceto­nitrile solvate.
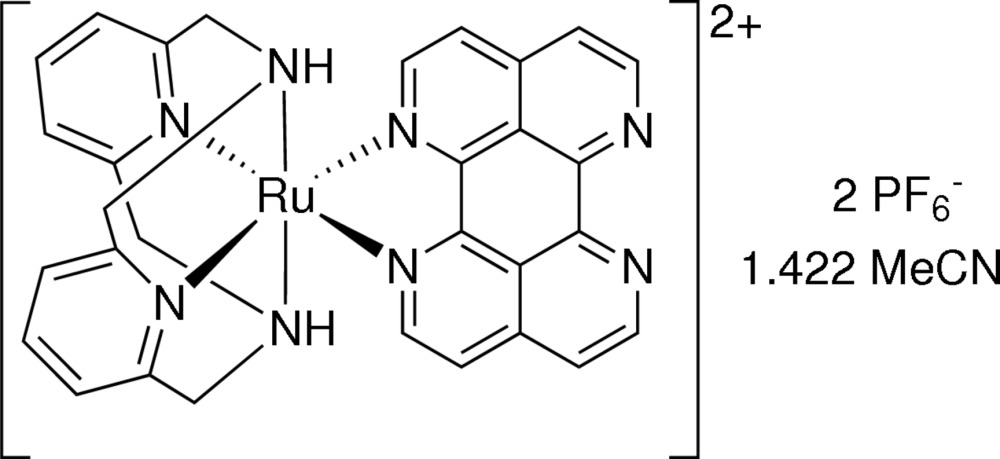


## Structural commentary   

**Figure 1:**
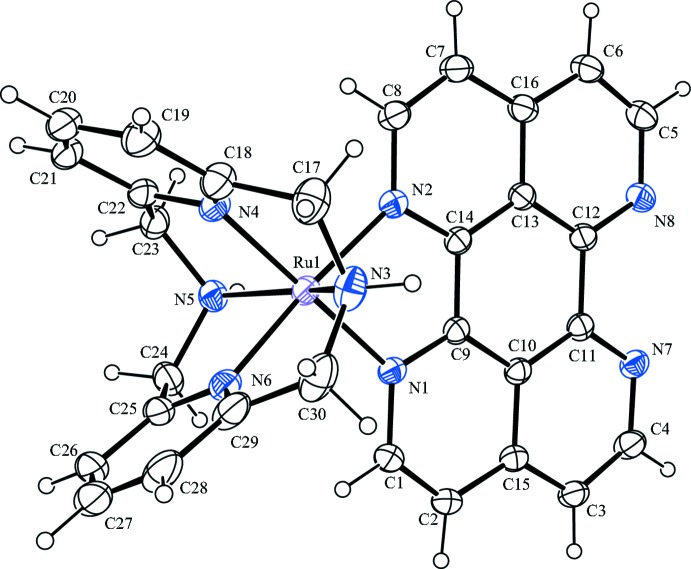
The mol­ecular structure of [Ru(L–N_4_H_2_)tape]^2+^ in [Ru(L–N_4_H_2_)tape](PF_6_)_2_·1.422CH_3_CN with the atomic numbering scheme and 30% probability displacement ellipsoids. Anions and solvent mol­ecules are omitted for clarity.

Fig. 1 ▶ illustrates the mol­ecular structure of the complex [Ru(L–N_4_H_2_)tape]^2+^ in [Ru(C_14_H_16_N_4_)(C_16_H_8_N_4_](PF_6_)_2_·1.422CH_3_CN. The Ru—N bond lengths formed by the tape ligand (Table 1 ▶) are very close to those reported earlier for [Ru(L–N_4_Me_2_)tape]^2+^ (Brietzke, Mickler, Kelling, Schilde *et al.*, 2012 ▶). The deviation of the N_amine_—Ru—N_amine_ angle [153.79 (10)°] from the idealized value of 180° is slightly larger than for analogous ruthenium L–N_4_Me_2_ complexes [155.46 (9)–155.93 (17)°; Brietzke, Mickler, Kelling, Schilde *et al.*, 2012 ▶].

## Supra­molecular features   

**Figure 2:**
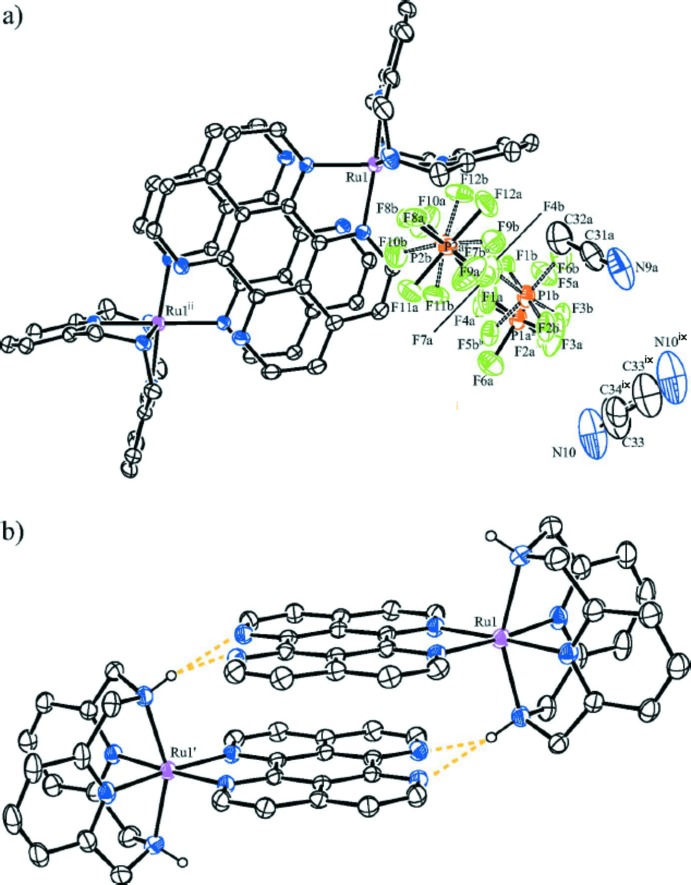
(*a*) Illustration of the asymmetric unit rendering the disorder of the hexa­fluorido­phosphate anions and aceto­nitrile solvate mol­ecules (see *Refinement* section for details). An additional π–π stacked [Ru(L–N_4_H_2_)tape]^2+^ cation demonstrates, due to the view along the normal of the tape ligand’s r.m.s. plane, the nearly face-to-face π–π stacking motif between the tape ligand moieties. The atomic numbering is shown for the anions and solvent mol­ecules as well as for the ruthenium atoms. Hydrogen atoms are omitted for clarity. [Symmetry codes: (ii) 1 − *x*, −*y*, 1 −*z*, (ix) 1 −*x*, 1 −*y*, −*z*.] (*b*) A side view of the dimer formed by two [Ru(L–N_4_H_2_)tape]^2+^ in [Ru(L–N_4_H_2_)tape](PF_6_)_2_·1.422CH_3_CN, featuring the stacking inter­actions *via* planar tape ligand moieties. Only H atoms essential for illustration of the hydrogen bonds, shown as orange dashed lines, are included.

**Figure 3:**
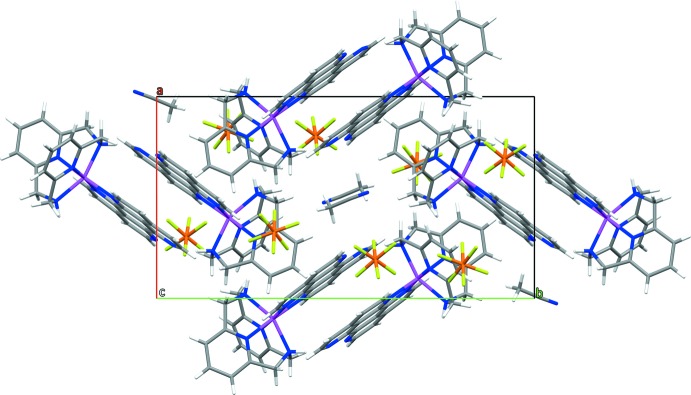
A packing diagram of the title compound is displayed along the *c* axis, illustrating the herringbone-type motif formed by two [Ru(L–N_4_H_2_)tape]^2+^ dimers. The disordered minor atoms are omitted for clarity.

In the crystal structure, the cations form discrete centrosymmetric dimers, similar to those seen previously in mononuclear ruthenium–tape complexes. The dimers are held together by π–π stacking inter­actions *via* the planar tetra­aza­perylene units, with a typical inter­planar distance of 3.39 Å. For the tape ligand, the root-mean-square deviation from planarity was calculated to be 0.0211 Å. However, in the case of [Ru(L–N_4_H_2_)tape]^2+^, the dimers are also connected through bifurcated hydrogen bonds between one of the two L–N_4_H_2_ ligand amine protons and both nitro­gen atoms of the non-coordin­ating tape ligand α,α′-di­imine unit of the second complex cation of the dimer. In the crystal structure, these additional hydrogen bonds result in a short Ru⋯Ru distance of 8.8306 (2) Å, a tape ligand centroid–centroid distance of 3.49 (2) Å and an angle of 13.7 (1.4)° between the ring normal and the centroid-to-centroid vector. Therefore, the π–π stacking motif can be described as parallel-displaced, but near to face-to-face (Fig. 2 ▶). In metal complexes, a near face-to-face alignment of the polycyclic units is extremely rare (Janiak, 2000 ▶). Furthermore, a large number of weak hydrogen bonds connect cations, anions and solvent mol­ecules, stabilizing the crystal packing (Table 2 ▶), supported by P—F⋯π-ring (tape, py) inter­actions with F⋯centroid distances from 2.925 to 3.984 Å. In the packing, the dimers are oriented in a herringbone-like motif, surrounded by hexa­fluorido­phosphate anions. The solvent aceto­nitrile mol­ecules fill the space between complex moieties (Fig. 3 ▶). For a description of the disorder of the anions and solvent mol­ecules, see the *Refinement* section.

## Database survey   

For related Ru^II^ complexes with 2,11-dimethyl-2,11-di­aza­[3.3](2,6)-pyridino­phane, see Brietzke, Mickler, Kelling, Schilde *et al.* (2012 ▶). For Ru^II^ tetra­aza­perylene complexes containing bi­pyridine-type ligands, see: Brietzke, Mickler, Kelling & Holdt (2012 ▶); Brietzke *et al.* (2014 ▶).

## Synthesis and crystallization   

The syntheses of the ligands L–N_4_H_2_ (Bottino *et al.*, 1988 ▶) and tape (Brietzke, Mickler, Kelling & Holdt, 2012 ▶) have been reported previously. [Ru(L–N_4_H_2_)tape](PF_6_)_2_ was synthesized as reported for [Ru(L–N_4_Me_2_)tape](PF_6_)_2_ (Brietzke, Mickler, Kelling, Schilde *et al.*, 2012 ▶), using L–N_4_H_2_ (73.5 mg, 306 µmol) instead of L–N_4_Me_2_. A yield of 44% (120.0 mg, 135 µmol) was obtained; m.p. > 573 K. ^1^H NMR = (MeCN–*d*_3_): δ = 8.69 (*d*, *J* = 5.5 Hz, 2H, C^*d*^—H), 8.56 (*d*, *J* = 6.6 Hz, 2H, C^*a*^—H), 8.01 (*t*, *J* = 8.0 Hz, 2H, C^4^—H), 7.77 (*d*, *J* = 5.5 Hz, 2H, C^*c*^—H), 7.72 (*d*, *J* = 6.6 Hz, 2H, C^*b*^—H), 7.65 (*d*, *J* = 8.0 Hz, 4H, C^3^—H + C^5^—H), 5.6 (*bs*, 2H, N—H), 4.83 (*bd*, J = 14.0 Hz, 4H, CH_2_), 4.47 (*d*, J = 17.4 Hz, 4H, CH_2_) p.p.m. ^13^C NMR = (MeCN-*d*_3_): δ = 160.0 (C^2^ + C^6^), 152.4 (C^*e*^), 150.28 (C^*d*^), 150.24 (C^*a*^), 145.7 (C^*f*^), 138.9 (C^4^), 136.7 (C^b′^), 123.5 (C^*b*^), 122.7 (C^3^ + C^5^), 122.0 (C^*c*^), 119.3 (C^e′^), 64.8 (CH_2_) p.p.m. ESI=-MS: calculated for [*M*–PF_6_]^+^ 743.0809; found 743.0778.

Crystals suitable for X-ray structure analysis were obtained by vapor diffusion of diethyl ether into a saturated aceto­nitrile solution of [Ru(L–N_4_H_2_)tape](PF_6_)_2_. The solution was filled into a test tube, which was placed into a diethyl ether-containing bottle. Dark-green crystals began to form at ambient temperature within a few days.

## Refinement   

Disorder was observed for both the hexa­fluorido­phosphate anions as well as the aceto­nitrile solvate mol­ecules. Both PF_6_ anions were refined as disordered over one major and one minor moiety each. The geometry of the minor moieties were each restrained to be similar to that of the major moieties (within an estimated standard deviation of 0.02 Å). The minor moieties were subjected to a rigid bond restraint (RIGU command of *SHELX2014*, estimated standard deviation 0.004 Å^2^), and the anisotropic displacement parameters of the major and minor phospho­rus atoms were each constrained to be identical. Associated with the major moiety of the PF_6_ anion of P1 is an aceto­nitrile mol­ecule that is absent for the minor moiety. Subject to the restraints and constraints used, the occupancy ratios refined to 0.9215 (17) to 0.0785 (17) for the PF_6_ units of P1*A* and P1*B*, and to 0.801 (6) and 0.199 (6) for those of P2*A* and P2*B*.

A second aceto­nitrile mol­ecule is disordered across a crystallographic inversion center, with substantial overlap for the two carbon atoms of symmetry-related mol­ecules. The geometry of the mol­ecule was restrained to be similar to that of the first aceto­nitrile mol­ecule, and the ADPs of its C and N atoms were restrained to be have similar *U*_ij_ components to their neighbors closer than 2 Å, including those of symmetry-related atoms (SIMU restraint in *SHELX2014*, estimated standard deviation 0.01 Å^2^).

All hydrogen atoms connected to C and N atoms were placed in their expected calculated positions and refined as riding with C—H = 0.98 (CH_3_), 0.99 (CH_2_), 0.95 (C_arom_), N—H = 1.0 Å, and with *U*_iso_(H) = 1.2*U*_eq_(C) with the exception of methyl hydrogen atoms, which were refined with *U*_iso_(H) = 1.5*U*_eq_(C).

Crystal data, data collection and structure refinement details are summarized in Table 3 ▶.

## Supplementary Material

Crystal structure: contains datablock(s) global, I. DOI: 10.1107/S1600536814021060/zl2602sup1.cif

Structure factors: contains datablock(s) I. DOI: 10.1107/S1600536814021060/zl2602Isup2.hkl

CCDC reference: 1025408

Additional supporting information:  crystallographic information; 3D view; checkCIF report

## Figures and Tables

**Table 1:** Selected bond lengths (Å)

Ru1—N6	2.005 (2)	Ru1—N2	2.055 (2)
Ru1—N4	2.018 (2)	Ru1—N5	2.132 (3)
Ru1—N1	2.045 (2)	Ru1—N3	2.135 (3)

**Table 2:** Hydrogen-bond geometry (Å, °)

*D*—H⋯*A*	*D*—H	H⋯*A*	*D*⋯*A*	*D*—H⋯*A*
N3—H3*N*⋯F2*A*_*a* ^i^	0.91	2.22	3.037 (4)	150
N3—H3*N*⋯F4*A*_*a* ^i^	0.91	2.55	3.236 (4)	133
N5—H5*N*⋯N7^ii^	0.88	2.20	3.006 (3)	153
N5—H5*N*⋯N8^ii^	0.88	2.69	3.373 (4)	135
C1—H1⋯F11*A*_*a* ^iii^	0.95	2.48	3.376 (5)	157
C1—H1⋯F11*B*_*b* ^iii^	0.95	2.28	3.019 (11)	134
C3—H3⋯F8*A*_*a* ^iv^	0.95	2.51	3.301 (8)	141
C3—H3⋯F12*A*_*a* ^iv^	0.95	2.60	3.414 (5)	144
C3—H3⋯F8*B*_*b* ^iv^	0.95	2.50	3.28 (3)	140
C3—H3⋯F12*B*_*b* ^iv^	0.95	2.37	3.283 (10)	161
C5—H5⋯F7*A*_*a* ^v^	0.95	2.50	3.404 (5)	159
C8—H8⋯F1*A*_*a*	0.95	2.53	3.211 (4)	128
C8—H8⋯F4*A*_*a*	0.95	2.40	3.085 (4)	129
C8—H8⋯F1*B*_*b*	0.95	2.50	3.42 (2)	163
C17—H17*A*⋯N9_*a* ^vi^	0.99	2.65	3.500 (8)	145
C17—H17*B*⋯F4*B*_*b*	0.99	2.34	3.15 (2)	138
C19—H19⋯F9*B*_*b* ^vi^	0.95	2.61	3.287 (12)	128
C21—H21⋯F11*A*_*a* ^vii^	0.95	2.48	3.166 (4)	129
C23—H23*B*⋯F6*A*_*a* ^iii^	0.99	2.51	3.409 (4)	151
C24—H24*A*⋯F7*A*_*a* ^iii^	0.99	2.53	3.224 (5)	127
C24—H24*B*⋯F2*B*_*b* ^iii^	0.99	2.09	3.00 (2)	152
C24—H24*B*⋯F5*B*_*b* ^iii^	0.99	2.51	3.41 (3)	150
C26—H26⋯F1*A*_*a* ^iii^	0.95	2.55	3.328 (5)	140
C26—H26⋯F4*B*_*b* ^iii^	0.95	2.36	3.26 (3)	156
C28—H28⋯N10^viii^	0.95	2.23	3.169 (12)	169
C30—H30*A*⋯N9_*a* ^vi^	0.99	2.59	3.484 (7)	151
C30—H30*B*⋯F4*A*_*a* ^i^	0.99	2.54	3.182 (5)	122
C32_*a*—H32*A*_*a*⋯F12*A*_*a* ^vi^	0.98	2.36	3.273 (8)	155
C32_*a*—H32*B*_*a*⋯F7*A*_*a*	0.98	2.54	3.150 (7)	121
C32_*a*—H32*C*_*a*⋯F1*A*_*a*	0.98	2.58	3.446 (8)	148

Symmetry codes: (i) 
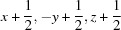
; (ii) 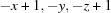; (iii) 
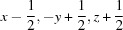
; (iv) 
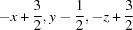
; (v) 
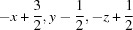
; (vi) 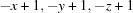; (vii) 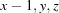; (viii) 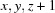.

**Table 3:** Experimental details

Crystal data	
Chemical formula	[Ru(C_14_H_16_N_4_)(C_16_H_8_N_4_)](PF_6_)_2_·1.422C_2_H_3_N
*M*_r_	946.11
Crystal system, space group	Monoclinic, *P*2_1_/*n*
Temperature (K)	150
*a*, *b*, *c* (Å)	11.7027 (3), 21.7157 (7), 13.9377 (4)
β (°)	97.938 (2)
*V* (Å^3^)	3508.08 (18)
*Z*	4
Radiation type	Mo *K*α
μ (mm^−1^)	0.65
Crystal size (mm)	1.30 × 0.65 × 0.31

Data collection	
Diffractometer	STOE IPDS 2
Absorption correction	Integration (*X-RED*; Stoe & Cie, 2011 ▶)
*T*_min_, *T*_max_	0.613, 0.843
No. of measured, independent and observed [*I* > 2σ(*I*)] reflections	60767, 9454, 7744
*R*_int_	0.087
(sin θ/λ)_max_ (Å^−1^)	0.689

Refinement	
*R*[*F* ^2^ > 2σ(*F* ^2^)], *wR*(*F* ^2^), *S*	0.048, 0.146, 1.09
No. of reflections	9454
No. of parameters	651
No. of restraints	183
H-atom treatment	H-atom parameters constrained
Δρ_max_, Δρ_min_ (e Å^−3^)	1.84, −1.22

Computer programs: *X-AREA* and *X-RED* (Stoe & Cie, 2011 ▶), *SHELXS97* and *SHELXL2014* (Sheldrick, 2008 ▶), *Mercury* (Macrae *et al.*, 2006 ▶), *ORTEP-3 for Windows* (Farrugia, 2012 ▶), *SHELXLE* (Hübschle *et al.*, 2011 ▶) and *publCIF* (Westrip, 2010 ▶).

## supplementary crystallographic information

### Crystal data

**Table:**

[Ru(C_14_H_16_N_4_)(C_16_H_8_N_4_)](PF_6_)_2_·1.422C_2_H_3_N	*F*(000) = 1893.4
*M**_r_* = 946.11	*D*_x_ = 1.791 Mg m^−^^3^
Monoclinic, *P*2_1_/*n*	Mo *K*α radiation, λ = 0.71073 Å
*a* = 11.7027 (3) Å	Cell parameters from 63561 reflections
*b* = 21.7157 (7) Å	θ = 1.5–29.6°
*c* = 13.9377 (4) Å	µ = 0.65 mm^−^^1^
β = 97.938 (2)°	*T* = 150 K
*V* = 3508.08 (18) Å^3^	Prism, dark green
*Z* = 4	1.30 × 0.65 × 0.31 mm

### Data collection

**Table:**

STOE IPDS 2 diffractometer	9454 independent reflections
Radiation source: sealed X-ray tube	7744 reflections with *I* > 2σ(*I*)
Plane graphite monochromator	*R*_int_ = 0.087
Detector resolution: 6.67 pixels mm^-1^	θ_max_ = 29.3°, θ_min_ = 1.8°
rotation method scans	*h* = −16→14
Absorption correction: integration (*X-RED*; Stoe & Cie, 2011)	*k* = −29→29
*T*_min_ = 0.613, *T*_max_ = 0.843	*l* = −19→19
60767 measured reflections	

### Refinement

**Table:**

Refinement on *F*^2^	Secondary atom site location: difference Fourier map
Least-squares matrix: full	Hydrogen site location: mixed
*R*[*F*^2^ > 2σ(*F*^2^)] = 0.048	H-atom parameters constrained
*wR*(*F*^2^) = 0.146	*w* = 1/[σ^2^(*F*_o_^2^) + (0.0787*P*)^2^ + 2.127*P*] where *P* = (*F*_o_^2^ + 2*F*_c_^2^)/3
*S* = 1.09	(Δ/σ)_max_ = 0.002
9454 reflections	Δρ_max_ = 1.84 e Å^−^^3^
651 parameters	Δρ_min_ = −1.22 e Å^−^^3^
183 restraints	Extinction correction: *SHELXL*, Fc^*^=kFc[1+0.001xFc^2^λ^3^/sin(2θ)]^-1/4^
Primary atom site location: structure-invariant direct methods	Extinction coefficient: 0.0041 (4)

### Special details

**Table:**

Geometry. All esds (except the esd in the dihedral angle between two l.s. planes) are estimated using the full covariance matrix. The cell esds are taken into account individually in the estimation of esds in distances, angles and torsion angles; correlations between esds in cell parameters are only used when they are defined by crystal symmetry. An approximate (isotropic) treatment of cell esds is used for estimating esds involving l.s. planes.

### Fractional atomic coordinates and isotropic or equivalent isotropic displacement parameters (Å^2^)

**Table:**

	*x*	*y*	*z*	*U*_iso_*/*U*_eq_	Occ. (<1)
Ru1	0.40042 (2)	0.19157 (2)	0.58944 (2)	0.03723 (9)	
N1	0.49654 (19)	0.11825 (11)	0.64626 (15)	0.0367 (5)	
N2	0.46354 (19)	0.15956 (11)	0.46830 (16)	0.0368 (4)	
N3	0.5182 (2)	0.26652 (13)	0.6188 (2)	0.0537 (7)	
H3N	0.5905	0.2585	0.6061	0.064*	
N4	0.3005 (2)	0.26143 (11)	0.53082 (16)	0.0398 (5)	
N5	0.2354 (2)	0.14885 (11)	0.58215 (17)	0.0411 (5)	
H5N	0.2357	0.1112	0.5582	0.049*	
N6	0.3522 (2)	0.22164 (12)	0.71383 (17)	0.0458 (6)	
N7	0.7375 (2)	−0.04526 (12)	0.55594 (17)	0.0410 (5)	
N8	0.7102 (2)	0.00109 (12)	0.36755 (17)	0.0431 (5)	
C1	0.5101 (2)	0.09624 (14)	0.73954 (19)	0.0419 (6)	
H1	0.4729	0.1176	0.7861	0.050*	
C2	0.5738 (2)	0.04565 (14)	0.76894 (18)	0.0403 (6)	
H2	0.5793	0.0321	0.8342	0.048*	
C3	0.7009 (3)	−0.03870 (14)	0.72236 (19)	0.0416 (6)	
H3	0.7137	−0.0553	0.7860	0.050*	
C4	0.7498 (3)	−0.06536 (15)	0.6482 (2)	0.0464 (7)	
H4	0.7961	−0.1009	0.6634	0.056*	
C5	0.6950 (3)	0.02554 (16)	0.2771 (2)	0.0489 (7)	
H5	0.7336	0.0061	0.2296	0.059*	
C6	0.6285 (3)	0.07603 (15)	0.2488 (2)	0.0465 (7)	
H6	0.6210	0.0904	0.1839	0.056*	
C7	0.4978 (3)	0.15793 (14)	0.3018 (2)	0.0435 (6)	
H7	0.4836	0.1756	0.2390	0.052*	
C8	0.4473 (3)	0.18253 (14)	0.3751 (2)	0.0433 (6)	
H8	0.3983	0.2173	0.3615	0.052*	
C9	0.5501 (2)	0.08765 (12)	0.58204 (17)	0.0330 (5)	
C10	0.6189 (2)	0.03545 (12)	0.60524 (17)	0.0327 (5)	
C11	0.6734 (2)	0.00476 (12)	0.53437 (18)	0.0339 (5)	
C12	0.6582 (2)	0.02915 (12)	0.43437 (18)	0.0349 (5)	
C13	0.5882 (2)	0.08220 (13)	0.41366 (17)	0.0341 (5)	
C14	0.5334 (2)	0.11075 (12)	0.48493 (18)	0.0335 (5)	
C15	0.6323 (2)	0.01304 (13)	0.70162 (18)	0.0357 (5)	
C16	0.5714 (2)	0.10613 (14)	0.31837 (19)	0.0392 (6)	
C17	0.4761 (3)	0.31805 (17)	0.5503 (3)	0.0596 (9)	
H17A	0.5040	0.3580	0.5787	0.072*	
H17B	0.5074	0.3126	0.4884	0.072*	
C18	0.3465 (3)	0.31816 (15)	0.5318 (3)	0.0486 (7)	
C19	0.2756 (3)	0.36940 (16)	0.5145 (2)	0.0534 (7)	
H19	0.3077	0.4096	0.5146	0.064*	
C20	0.1575 (3)	0.36070 (16)	0.4970 (2)	0.0499 (7)	
H20	0.1076	0.3952	0.4850	0.060*	
C21	0.1115 (3)	0.30167 (15)	0.4970 (2)	0.0467 (7)	
H21	0.0304	0.2953	0.4854	0.056*	
C22	0.1864 (2)	0.25246 (14)	0.51419 (18)	0.0406 (6)	
C23	0.1520 (3)	0.18614 (14)	0.5144 (2)	0.0447 (6)	
H23A	0.1483	0.1693	0.4480	0.054*	
H23B	0.0741	0.1828	0.5340	0.054*	
C24	0.2034 (3)	0.14619 (16)	0.6821 (2)	0.0505 (7)	
H24A	0.2333	0.1076	0.7143	0.061*	
H24B	0.1183	0.1462	0.6788	0.061*	
C25	0.2531 (3)	0.20074 (15)	0.7401 (2)	0.0486 (7)	
C26	0.2081 (4)	0.22746 (19)	0.8173 (2)	0.0605 (10)	
H26	0.1382	0.2129	0.8367	0.073*	
C27	0.2687 (4)	0.2763 (2)	0.8653 (2)	0.0725 (13)	
H27	0.2399	0.2952	0.9186	0.087*	
C28	0.3680 (4)	0.2973 (2)	0.8372 (3)	0.0711 (13)	
H28	0.4082	0.3308	0.8703	0.085*	
C29	0.4110 (3)	0.26922 (17)	0.7592 (2)	0.0578 (9)	
C30	0.5223 (3)	0.2836 (2)	0.7230 (3)	0.0664 (11)	
H30A	0.5388	0.3281	0.7309	0.080*	
H30B	0.5856	0.2607	0.7620	0.080*	
P2A_a	0.8068 (3)	0.42105 (13)	0.4826 (2)	0.0447 (4)	0.801 (6)
F7A_a	0.7169 (3)	0.4256 (2)	0.3859 (2)	0.0686 (11)	0.801 (6)
F8A_a	0.8952 (6)	0.4132 (4)	0.5788 (4)	0.073 (2)	0.801 (6)
F9A_a	0.8867 (5)	0.4699 (2)	0.4427 (3)	0.119 (2)	0.801 (6)
F10A_a	0.7290 (3)	0.3697 (2)	0.5217 (3)	0.1071 (18)	0.801 (6)
F11A_a	0.8748 (3)	0.3704 (2)	0.4319 (2)	0.0715 (11)	0.801 (6)
F12A_a	0.7436 (5)	0.4740 (2)	0.5315 (3)	0.122 (2)	0.801 (6)
P2B_b	0.7944 (11)	0.4130 (6)	0.4882 (10)	0.0447 (4)	0.199 (6)
F7B_b	0.6911 (14)	0.4026 (9)	0.4033 (11)	0.076 (4)	0.199 (6)
F8B_b	0.8948 (19)	0.4222 (13)	0.5756 (15)	0.053 (4)	0.199 (6)
F9B_b	0.7949 (14)	0.4848 (5)	0.4693 (10)	0.075 (4)	0.199 (6)
F10B_b	0.8082 (15)	0.3414 (5)	0.4931 (10)	0.090 (4)	0.199 (6)
F11B_b	0.8886 (12)	0.4117 (9)	0.4167 (9)	0.081 (4)	0.199 (6)
F12B_b	0.6988 (9)	0.4239 (8)	0.5536 (8)	0.068 (3)	0.199 (6)
P1A_a	0.33081 (8)	0.31111 (5)	0.17529 (8)	0.0538 (3)	0.9215 (17)
F1A_a	0.4320 (3)	0.31248 (12)	0.2642 (3)	0.0900 (11)	0.9215 (17)
F2A_a	0.2279 (2)	0.30908 (12)	0.08663 (17)	0.0644 (6)	0.9215 (17)
F3A_a	0.3730 (4)	0.37384 (18)	0.1391 (4)	0.1430 (19)	0.9215 (17)
F4A_a	0.2860 (2)	0.24711 (14)	0.2119 (2)	0.0810 (8)	0.9215 (17)
F5A_a	0.2477 (3)	0.3457 (2)	0.2363 (2)	0.1186 (15)	0.9215 (17)
F6A_a	0.4133 (3)	0.2749 (2)	0.1160 (3)	0.1041 (11)	0.9215 (17)
P1B_b	0.3791 (8)	0.3534 (5)	0.2328 (8)	0.0538 (3)	0.0785 (17)
F1B_b	0.2932 (17)	0.3068 (10)	0.2780 (18)	0.061 (5)	0.0785 (17)
F2B_b	0.4610 (19)	0.3981 (11)	0.185 (2)	0.082 (6)	0.0785 (17)
F3B_b	0.2821 (18)	0.3626 (12)	0.1461 (17)	0.073 (5)	0.0785 (17)
F4B_b	0.4705 (19)	0.3486 (12)	0.3286 (16)	0.076 (5)	0.0785 (17)
F5B_b	0.433 (2)	0.2970 (9)	0.187 (2)	0.065 (5)	0.0785 (17)
F6B_b	0.324 (2)	0.4083 (11)	0.288 (2)	0.085 (6)	0.0785 (17)
N9_a	0.5253 (5)	0.5581 (3)	0.2892 (6)	0.135 (3)	0.9215 (17)
C31_a	0.4952 (4)	0.5135 (3)	0.3184 (5)	0.0832 (15)	0.9215 (17)
C32_a	0.4527 (5)	0.4596 (3)	0.3578 (6)	0.105 (2)	0.9215 (17)
H32A_a	0.3799	0.4689	0.3821	0.158*	0.9215 (17)
H32B_a	0.5092	0.4446	0.4111	0.158*	0.9215 (17)
H32C_a	0.4394	0.4279	0.3074	0.158*	0.9215 (17)
N10	0.4690 (12)	0.4178 (5)	−0.0541 (15)	0.189 (6)	0.5
C33	0.482 (2)	0.4656 (7)	−0.023 (2)	0.151 (6)	0.5
C34	0.5079 (14)	0.5265 (5)	0.0086 (15)	0.088 (4)	0.5
H34A	0.4426	0.5534	−0.0144	0.132*	0.5
H34B	0.5769	0.5410	−0.0172	0.132*	0.5
H34C	0.5219	0.5273	0.0796	0.132*	0.5

### Atomic displacement parameters (Å^2^)

**Table:**

	*U*^11^	*U*^22^	*U*^33^	*U*^12^	*U*^13^	*U*^23^
Ru1	0.03631 (13)	0.04128 (15)	0.03304 (13)	0.00739 (8)	0.00104 (8)	−0.00181 (8)
N1	0.0365 (11)	0.0423 (12)	0.0302 (10)	0.0077 (9)	0.0011 (8)	−0.0006 (8)
N2	0.0355 (10)	0.0411 (12)	0.0333 (10)	0.0032 (9)	0.0029 (8)	0.0002 (9)
N3	0.0371 (12)	0.0515 (15)	0.0704 (18)	0.0019 (11)	−0.0005 (12)	−0.0090 (13)
N4	0.0406 (12)	0.0421 (12)	0.0352 (10)	0.0084 (10)	−0.0001 (9)	0.0012 (9)
N5	0.0420 (12)	0.0401 (12)	0.0417 (12)	0.0047 (10)	0.0078 (9)	−0.0039 (9)
N6	0.0511 (14)	0.0498 (14)	0.0344 (11)	0.0186 (11)	−0.0018 (10)	−0.0052 (10)
N7	0.0402 (12)	0.0447 (13)	0.0363 (11)	0.0072 (10)	−0.0011 (9)	−0.0051 (9)
N8	0.0434 (12)	0.0516 (14)	0.0348 (11)	0.0036 (10)	0.0076 (9)	−0.0061 (10)
C1	0.0432 (14)	0.0517 (16)	0.0308 (12)	0.0084 (12)	0.0055 (10)	−0.0032 (11)
C2	0.0434 (14)	0.0486 (15)	0.0282 (11)	0.0044 (12)	0.0024 (10)	0.0005 (10)
C3	0.0447 (14)	0.0463 (15)	0.0318 (12)	0.0063 (12)	−0.0018 (10)	−0.0003 (10)
C4	0.0505 (16)	0.0466 (16)	0.0394 (14)	0.0125 (13)	−0.0029 (12)	−0.0025 (12)
C5	0.0502 (16)	0.0620 (19)	0.0362 (13)	0.0018 (14)	0.0122 (12)	−0.0040 (13)
C6	0.0510 (16)	0.0565 (18)	0.0329 (12)	−0.0012 (14)	0.0093 (11)	0.0022 (12)
C7	0.0464 (15)	0.0485 (16)	0.0349 (12)	−0.0006 (12)	0.0029 (11)	0.0080 (11)
C8	0.0422 (14)	0.0469 (15)	0.0394 (14)	0.0027 (12)	0.0010 (11)	0.0070 (11)
C9	0.0286 (10)	0.0381 (13)	0.0316 (11)	0.0003 (9)	0.0015 (9)	−0.0022 (9)
C10	0.0302 (11)	0.0356 (12)	0.0311 (11)	−0.0008 (9)	0.0000 (9)	−0.0022 (9)
C11	0.0300 (11)	0.0376 (12)	0.0331 (11)	−0.0031 (9)	0.0006 (9)	−0.0044 (9)
C12	0.0321 (11)	0.0410 (13)	0.0312 (11)	−0.0022 (10)	0.0033 (9)	−0.0039 (10)
C13	0.0305 (11)	0.0400 (13)	0.0314 (11)	−0.0034 (9)	0.0026 (9)	−0.0015 (9)
C14	0.0296 (11)	0.0380 (12)	0.0321 (11)	−0.0008 (9)	0.0015 (9)	−0.0012 (9)
C15	0.0353 (12)	0.0402 (13)	0.0303 (11)	0.0002 (10)	0.0006 (9)	−0.0018 (10)
C16	0.0389 (13)	0.0461 (15)	0.0327 (12)	−0.0044 (11)	0.0053 (10)	0.0006 (10)
C17	0.0472 (17)	0.0492 (18)	0.081 (3)	−0.0014 (14)	0.0055 (17)	0.0021 (17)
C18	0.0466 (16)	0.0450 (15)	0.0534 (17)	0.0032 (13)	0.0043 (13)	0.0038 (13)
C19	0.0590 (19)	0.0432 (16)	0.0572 (18)	0.0068 (14)	0.0060 (15)	0.0073 (14)
C20	0.0555 (18)	0.0518 (17)	0.0414 (14)	0.0147 (14)	0.0025 (13)	0.0042 (13)
C21	0.0440 (15)	0.0586 (18)	0.0354 (13)	0.0153 (13)	−0.0018 (11)	−0.0015 (12)
C22	0.0407 (13)	0.0492 (15)	0.0303 (11)	0.0089 (12)	−0.0002 (10)	−0.0032 (10)
C23	0.0376 (14)	0.0517 (17)	0.0433 (15)	0.0064 (12)	0.0005 (11)	−0.0073 (12)
C24	0.0557 (18)	0.0502 (17)	0.0493 (16)	0.0112 (14)	0.0208 (14)	0.0039 (13)
C25	0.0599 (18)	0.0540 (17)	0.0324 (13)	0.0250 (14)	0.0081 (12)	0.0026 (11)
C26	0.074 (2)	0.072 (2)	0.0370 (14)	0.0348 (19)	0.0137 (14)	0.0020 (14)
C27	0.088 (3)	0.088 (3)	0.0377 (15)	0.049 (2)	−0.0052 (17)	−0.0161 (17)
C28	0.072 (3)	0.079 (3)	0.0531 (19)	0.037 (2)	−0.0241 (18)	−0.0289 (18)
C29	0.0607 (19)	0.0594 (19)	0.0465 (16)	0.0259 (16)	−0.0171 (14)	−0.0149 (14)
C30	0.0500 (18)	0.068 (2)	0.074 (2)	0.0122 (17)	−0.0178 (17)	−0.0264 (19)
P2A_a	0.0495 (8)	0.0536 (9)	0.0305 (5)	0.0068 (6)	0.0035 (5)	−0.0021 (6)
F7A_a	0.0612 (19)	0.095 (3)	0.0446 (14)	0.0162 (18)	−0.0104 (13)	0.0000 (16)
F8A_a	0.074 (3)	0.103 (5)	0.038 (2)	0.017 (3)	−0.0093 (19)	−0.001 (2)
F9A_a	0.144 (4)	0.119 (4)	0.087 (3)	−0.066 (3)	−0.012 (3)	0.035 (2)
F10A_a	0.065 (2)	0.146 (4)	0.111 (3)	−0.030 (2)	0.017 (2)	0.056 (3)
F11A_a	0.0552 (16)	0.095 (3)	0.0623 (18)	0.0215 (17)	−0.0006 (13)	−0.0264 (18)
F12A_a	0.166 (5)	0.118 (4)	0.070 (2)	0.093 (4)	−0.020 (3)	−0.035 (2)
P2B_b	0.0495 (8)	0.0536 (9)	0.0305 (5)	0.0068 (6)	0.0035 (5)	−0.0021 (6)
F7B_b	0.075 (7)	0.082 (9)	0.067 (7)	0.024 (5)	−0.008 (5)	−0.032 (6)
F8B_b	0.056 (7)	0.062 (8)	0.042 (7)	−0.006 (5)	0.008 (5)	−0.015 (5)
F9B_b	0.101 (8)	0.057 (5)	0.070 (7)	0.003 (4)	0.015 (6)	−0.008 (4)
F10B_b	0.115 (10)	0.067 (5)	0.080 (7)	0.016 (5)	−0.020 (6)	−0.016 (4)
F11B_b	0.085 (7)	0.110 (10)	0.050 (5)	0.029 (6)	0.021 (5)	−0.011 (6)
F12B_b	0.052 (5)	0.097 (9)	0.056 (5)	−0.012 (5)	0.011 (4)	−0.038 (5)
P1A_a	0.0385 (4)	0.0567 (6)	0.0637 (6)	0.0047 (4)	−0.0018 (4)	0.0080 (4)
F1A_a	0.0674 (17)	0.0674 (17)	0.119 (3)	−0.0021 (13)	−0.0435 (18)	0.0102 (16)
F2A_a	0.0545 (13)	0.0872 (18)	0.0500 (12)	0.0095 (11)	0.0013 (10)	0.0056 (10)
F3A_a	0.107 (3)	0.093 (2)	0.210 (5)	−0.032 (2)	−0.046 (3)	0.080 (3)
F4A_a	0.0538 (13)	0.0899 (19)	0.0937 (19)	−0.0141 (13)	−0.0099 (13)	0.0333 (16)
F5A_a	0.104 (2)	0.160 (4)	0.082 (2)	0.069 (3)	−0.0224 (18)	−0.049 (2)
F6A_a	0.0612 (17)	0.136 (3)	0.121 (3)	0.0272 (19)	0.0349 (17)	0.007 (2)
P1B_b	0.0385 (4)	0.0567 (6)	0.0637 (6)	0.0047 (4)	−0.0018 (4)	0.0080 (4)
F1B_b	0.040 (7)	0.061 (9)	0.078 (10)	0.011 (6)	−0.008 (7)	0.022 (7)
F2B_b	0.054 (9)	0.066 (9)	0.123 (12)	0.009 (7)	0.004 (8)	0.029 (8)
F3B_b	0.050 (8)	0.063 (11)	0.103 (8)	0.001 (7)	−0.004 (6)	0.033 (7)
F4B_b	0.052 (8)	0.066 (11)	0.103 (8)	0.006 (7)	−0.014 (6)	0.008 (7)
F5B_b	0.047 (9)	0.058 (7)	0.087 (10)	0.002 (6)	−0.001 (8)	0.020 (7)
F6B_b	0.061 (10)	0.073 (8)	0.119 (11)	0.012 (7)	0.002 (8)	0.005 (7)
N9_a	0.069 (3)	0.072 (3)	0.264 (9)	0.004 (3)	0.021 (4)	−0.011 (4)
C31_a	0.047 (2)	0.073 (3)	0.127 (5)	0.007 (2)	0.005 (3)	−0.018 (3)
C32_a	0.064 (3)	0.099 (4)	0.152 (6)	0.001 (3)	0.010 (3)	0.007 (4)
N10	0.114 (9)	0.095 (8)	0.335 (18)	−0.005 (7)	−0.052 (11)	−0.012 (11)
C33	0.079 (9)	0.113 (10)	0.247 (15)	−0.004 (9)	−0.025 (10)	−0.005 (11)
C34	0.039 (5)	0.085 (6)	0.142 (10)	0.010 (5)	0.018 (6)	0.025 (7)

### Geometric parameters (Å, º)

**Table:**

Ru1—N6	2.005 (2)	C20—C21	1.390 (5)
Ru1—N4	2.018 (2)	C20—H20	0.9500
Ru1—N1	2.045 (2)	C21—C22	1.382 (4)
Ru1—N2	2.055 (2)	C21—H21	0.9500
Ru1—N5	2.132 (3)	C22—C23	1.495 (4)
Ru1—N3	2.135 (3)	C23—H23A	0.9900
N1—C9	1.338 (3)	C23—H23B	0.9900
N1—C1	1.374 (3)	C24—C25	1.505 (5)
N2—C14	1.339 (3)	C24—H24A	0.9900
N2—C8	1.380 (4)	C24—H24B	0.9900
N3—C30	1.492 (5)	C25—C26	1.388 (4)
N3—C17	1.509 (5)	C26—C27	1.395 (6)
N3—H3N	0.9051	C26—H26	0.9500
N4—C22	1.338 (4)	C27—C28	1.356 (7)
N4—C18	1.343 (4)	C27—H27	0.9500
N5—C24	1.493 (4)	C28—C29	1.400 (5)
N5—C23	1.499 (4)	C28—H28	0.9500
N5—H5N	0.8825	C29—C30	1.494 (6)
N6—C25	1.342 (5)	C30—H30A	0.9900
N6—C29	1.349 (5)	C30—H30B	0.9900
N7—C11	1.330 (4)	P2A_a—F9A_a	1.566 (4)
N7—C4	1.347 (4)	P2A_a—F12A_a	1.573 (4)
N8—C12	1.328 (3)	P2A_a—F11A_a	1.581 (4)
N8—C5	1.356 (4)	P2A_a—F10A_a	1.583 (4)
C1—C2	1.359 (4)	P2A_a—F8A_a	1.585 (4)
C1—H1	0.9500	P2A_a—F7A_a	1.594 (4)
C2—C15	1.424 (4)	P2B_b—F12B_b	1.556 (13)
C2—H2	0.9500	P2B_b—F10B_b	1.565 (14)
C3—C4	1.376 (4)	P2B_b—F9B_b	1.581 (14)
C3—C15	1.388 (4)	P2B_b—F8B_b	1.584 (14)
C3—H3	0.9500	P2B_b—F11B_b	1.585 (13)
C4—H4	0.9500	P2B_b—F7B_b	1.586 (14)
C5—C6	1.371 (5)	P1A_a—F3A_a	1.556 (3)
C5—H5	0.9500	P1A_a—F6A_a	1.566 (3)
C6—C16	1.411 (4)	P1A_a—F5A_a	1.569 (3)
C6—H6	0.9500	P1A_a—F1A_a	1.592 (3)
C7—C8	1.358 (4)	P1A_a—F4A_a	1.594 (3)
C7—C16	1.416 (4)	P1A_a—F2A_a	1.603 (3)
C7—H7	0.9500	P1B_b—F3B_b	1.552 (15)
C8—H8	0.9500	P1B_b—F5B_b	1.555 (16)
C9—C10	1.402 (4)	P1B_b—F2B_b	1.576 (15)
C9—C14	1.431 (3)	P1B_b—F4B_b	1.594 (15)
C10—C11	1.415 (3)	P1B_b—F6B_b	1.601 (16)
C10—C15	1.417 (3)	P1B_b—F1B_b	1.614 (15)
C11—C12	1.479 (4)	N9_a—C31_a	1.125 (8)
C12—C13	1.420 (4)	C31_a—C32_a	1.413 (8)
C13—C14	1.400 (3)	C32_a—H32A_a	0.9800
C13—C16	1.414 (4)	C32_a—H32B_a	0.9800
C17—C18	1.504 (5)	C32_a—H32C_a	0.9800
C17—H17A	0.9900	N10—C33	1.125 (8)
C17—H17B	0.9900	C33—C34	1.413 (8)
C18—C19	1.389 (5)	C34—H34A	0.9800
C19—C20	1.383 (5)	C34—H34B	0.9800
C19—H19	0.9500	C34—H34C	0.9800
			
N6—Ru1—N4	83.61 (9)	C21—C22—C23	125.5 (3)
N6—Ru1—N1	97.14 (9)	C22—C23—N5	111.7 (2)
N4—Ru1—N1	177.60 (10)	C22—C23—H23A	109.3
N6—Ru1—N2	175.16 (9)	N5—C23—H23A	109.3
N4—Ru1—N2	100.11 (9)	C22—C23—H23B	109.3
N1—Ru1—N2	79.27 (9)	N5—C23—H23B	109.3
N6—Ru1—N5	79.72 (10)	H23A—C23—H23B	107.9
N4—Ru1—N5	80.65 (10)	N5—C24—C25	110.0 (3)
N1—Ru1—N5	97.22 (9)	N5—C24—H24A	109.7
N2—Ru1—N5	103.86 (9)	C25—C24—H24A	109.7
N6—Ru1—N3	80.65 (12)	N5—C24—H24B	109.7
N4—Ru1—N3	80.06 (11)	C25—C24—H24B	109.7
N1—Ru1—N3	102.31 (11)	H24A—C24—H24B	108.2
N2—Ru1—N3	96.87 (11)	N6—C25—C26	120.3 (3)
N5—Ru1—N3	153.79 (10)	N6—C25—C24	113.8 (3)
C9—N1—C1	117.2 (2)	C26—C25—C24	125.8 (4)
C9—N1—Ru1	114.26 (17)	C25—C26—C27	117.8 (4)
C1—N1—Ru1	128.52 (18)	C25—C26—H26	121.1
C14—N2—C8	116.8 (2)	C27—C26—H26	121.1
C14—N2—Ru1	113.92 (17)	C28—C27—C26	121.2 (3)
C8—N2—Ru1	129.2 (2)	C28—C27—H27	119.4
C30—N3—C17	113.3 (3)	C26—C27—H27	119.4
C30—N3—Ru1	108.0 (2)	C27—C28—C29	119.4 (4)
C17—N3—Ru1	107.4 (2)	C27—C28—H28	120.3
C30—N3—H3N	109.4	C29—C28—H28	120.3
C17—N3—H3N	104.6	N6—C29—C28	118.9 (4)
Ru1—N3—H3N	114.3	N6—C29—C30	114.4 (3)
C22—N4—C18	121.6 (3)	C28—C29—C30	126.5 (4)
C22—N4—Ru1	118.0 (2)	N3—C30—C29	111.4 (3)
C18—N4—Ru1	118.4 (2)	N3—C30—H30A	109.3
C24—N5—C23	112.5 (2)	C29—C30—H30A	109.3
C24—N5—Ru1	108.35 (19)	N3—C30—H30B	109.3
C23—N5—Ru1	107.56 (18)	C29—C30—H30B	109.3
C24—N5—H5N	109.4	H30A—C30—H30B	108.0
C23—N5—H5N	107.3	F9A_a—P2A_a—F12A_a	89.9 (4)
Ru1—N5—H5N	111.7	F9A_a—P2A_a—F11A_a	87.2 (3)
C25—N6—C29	122.3 (3)	F12A_a—P2A_a—F11A_a	177.0 (4)
C25—N6—Ru1	118.9 (2)	F9A_a—P2A_a—F10A_a	177.8 (4)
C29—N6—Ru1	117.9 (2)	F12A_a—P2A_a—F10A_a	92.3 (4)
C11—N7—C4	117.6 (2)	F11A_a—P2A_a—F10A_a	90.7 (3)
C12—N8—C5	117.3 (3)	F9A_a—P2A_a—F8A_a	91.2 (4)
C2—C1—N1	123.6 (2)	F12A_a—P2A_a—F8A_a	90.2 (4)
C2—C1—H1	118.2	F11A_a—P2A_a—F8A_a	89.6 (3)
N1—C1—H1	118.2	F10A_a—P2A_a—F8A_a	88.4 (4)
C1—C2—C15	119.8 (2)	F9A_a—P2A_a—F7A_a	90.8 (3)
C1—C2—H2	120.1	F12A_a—P2A_a—F7A_a	91.5 (3)
C15—C2—H2	120.1	F11A_a—P2A_a—F7A_a	88.8 (2)
C4—C3—C15	118.2 (3)	F10A_a—P2A_a—F7A_a	89.5 (2)
C4—C3—H3	120.9	F8A_a—P2A_a—F7A_a	177.3 (4)
C15—C3—H3	120.9	F12B_b—P2B_b—F10B_b	101.7 (11)
N7—C4—C3	125.4 (3)	F12B_b—P2B_b—F9B_b	88.2 (10)
N7—C4—H4	117.3	F10B_b—P2B_b—F9B_b	170.0 (12)
C3—C4—H4	117.3	F12B_b—P2B_b—F8B_b	92.7 (12)
N8—C5—C6	125.2 (3)	F10B_b—P2B_b—F8B_b	91.7 (13)
N8—C5—H5	117.4	F9B_b—P2B_b—F8B_b	89.2 (12)
C6—C5—H5	117.4	F12B_b—P2B_b—F11B_b	171.9 (13)
C5—C6—C16	118.5 (3)	F10B_b—P2B_b—F11B_b	86.2 (11)
C5—C6—H6	120.8	F9B_b—P2B_b—F11B_b	83.9 (10)
C16—C6—H6	120.8	F8B_b—P2B_b—F11B_b	88.8 (12)
C8—C7—C16	120.6 (3)	F12B_b—P2B_b—F7B_b	85.6 (9)
C8—C7—H7	119.7	F10B_b—P2B_b—F7B_b	87.3 (10)
C16—C7—H7	119.7	F9B_b—P2B_b—F7B_b	92.1 (10)
C7—C8—N2	123.2 (3)	F8B_b—P2B_b—F7B_b	177.8 (14)
C7—C8—H8	118.4	F11B_b—P2B_b—F7B_b	93.0 (11)
N2—C8—H8	118.4	F3A_a—P1A_a—F6A_a	91.3 (3)
N1—C9—C10	123.7 (2)	F3A_a—P1A_a—F5A_a	90.3 (3)
N1—C9—C14	116.3 (2)	F6A_a—P1A_a—F5A_a	178.4 (3)
C10—C9—C14	120.0 (2)	F3A_a—P1A_a—F1A_a	90.06 (19)
C9—C10—C11	121.3 (2)	F6A_a—P1A_a—F1A_a	88.5 (2)
C9—C10—C15	118.8 (2)	F5A_a—P1A_a—F1A_a	91.1 (2)
C11—C10—C15	119.9 (2)	F3A_a—P1A_a—F4A_a	179.29 (18)
N7—C11—C10	121.4 (2)	F6A_a—P1A_a—F4A_a	89.1 (2)
N7—C11—C12	119.8 (2)	F5A_a—P1A_a—F4A_a	89.3 (2)
C10—C11—C12	118.8 (2)	F1A_a—P1A_a—F4A_a	90.53 (15)
N8—C12—C13	122.5 (2)	F3A_a—P1A_a—F2A_a	90.70 (18)
N8—C12—C11	119.3 (2)	F6A_a—P1A_a—F2A_a	91.83 (18)
C13—C12—C11	118.2 (2)	F5A_a—P1A_a—F2A_a	88.55 (15)
C14—C13—C16	119.1 (2)	F1A_a—P1A_a—F2A_a	179.18 (18)
C14—C13—C12	121.6 (2)	F4A_a—P1A_a—F2A_a	88.72 (14)
C16—C13—C12	119.3 (2)	F3B_b—P1B_b—F5B_b	94.2 (14)
N2—C14—C13	123.8 (2)	F3B_b—P1B_b—F2B_b	90.7 (13)
N2—C14—C9	116.2 (2)	F5B_b—P1B_b—F2B_b	90.3 (13)
C13—C14—C9	120.0 (2)	F3B_b—P1B_b—F4B_b	173.7 (15)
C3—C15—C10	117.4 (2)	F5B_b—P1B_b—F4B_b	91.9 (13)
C3—C15—C2	125.6 (2)	F2B_b—P1B_b—F4B_b	91.0 (13)
C10—C15—C2	117.0 (2)	F3B_b—P1B_b—F6B_b	88.9 (13)
C6—C16—C13	117.2 (3)	F5B_b—P1B_b—F6B_b	175.5 (15)
C6—C16—C7	126.2 (3)	F2B_b—P1B_b—F6B_b	92.9 (14)
C13—C16—C7	116.5 (2)	F4B_b—P1B_b—F6B_b	85.0 (13)
C18—C17—N3	110.1 (3)	F3B_b—P1B_b—F1B_b	87.5 (12)
C18—C17—H17A	109.6	F5B_b—P1B_b—F1B_b	88.8 (12)
N3—C17—H17A	109.6	F2B_b—P1B_b—F1B_b	178.0 (15)
C18—C17—H17B	109.6	F4B_b—P1B_b—F1B_b	90.9 (12)
N3—C17—H17B	109.6	F6B_b—P1B_b—F1B_b	88.1 (13)
H17A—C17—H17B	108.2	N9_a—C31_a—C32_a	176.6 (7)
N4—C18—C19	120.3 (3)	C31_a—C32_a—H32A_a	109.5
N4—C18—C17	113.2 (3)	C31_a—C32_a—H32B_a	109.5
C19—C18—C17	126.5 (3)	H32A_a—C32_a—H32B_a	109.5
C20—C19—C18	118.6 (3)	C31_a—C32_a—H32C_a	109.5
C20—C19—H19	120.7	H32A_a—C32_a—H32C_a	109.5
C18—C19—H19	120.7	H32B_a—C32_a—H32C_a	109.5
C19—C20—C21	120.3 (3)	N10—C33—C34	174.0 (19)
C19—C20—H20	119.9	C33—C34—H34A	109.5
C21—C20—H20	119.9	C33—C34—H34B	109.5
C22—C21—C20	118.4 (3)	H34A—C34—H34B	109.5
C22—C21—H21	120.8	C33—C34—H34C	109.5
C20—C21—H21	120.8	H34A—C34—H34C	109.5
N4—C22—C21	120.8 (3)	H34B—C34—H34C	109.5
N4—C22—C23	113.7 (2)		
			
C9—N1—C1—C2	−0.3 (4)	C1—C2—C15—C10	−0.9 (4)
Ru1—N1—C1—C2	177.9 (2)	C5—C6—C16—C13	0.7 (4)
N1—C1—C2—C15	1.0 (5)	C5—C6—C16—C7	−178.7 (3)
C11—N7—C4—C3	0.5 (5)	C14—C13—C16—C6	179.9 (3)
C15—C3—C4—N7	0.4 (5)	C12—C13—C16—C6	−1.4 (4)
C12—N8—C5—C6	−1.5 (5)	C14—C13—C16—C7	−0.7 (4)
N8—C5—C6—C16	0.8 (5)	C12—C13—C16—C7	178.1 (2)
C16—C7—C8—N2	0.0 (5)	C8—C7—C16—C6	179.6 (3)
C14—N2—C8—C7	0.3 (4)	C8—C7—C16—C13	0.2 (4)
Ru1—N2—C8—C7	177.6 (2)	C30—N3—C17—C18	−85.1 (4)
C1—N1—C9—C10	−0.5 (4)	Ru1—N3—C17—C18	34.0 (4)
Ru1—N1—C9—C10	−178.98 (19)	C22—N4—C18—C19	−0.6 (5)
C1—N1—C9—C14	179.0 (2)	Ru1—N4—C18—C19	−164.4 (3)
Ru1—N1—C9—C14	0.6 (3)	C22—N4—C18—C17	−179.1 (3)
N1—C9—C10—C11	179.9 (2)	Ru1—N4—C18—C17	17.1 (4)
C14—C9—C10—C11	0.3 (4)	N3—C17—C18—N4	−34.3 (4)
N1—C9—C10—C15	0.6 (4)	N3—C17—C18—C19	147.3 (3)
C14—C9—C10—C15	−179.0 (2)	N4—C18—C19—C20	0.5 (5)
C4—N7—C11—C10	−0.8 (4)	C17—C18—C19—C20	178.8 (3)
C4—N7—C11—C12	179.3 (3)	C18—C19—C20—C21	0.0 (5)
C9—C10—C11—N7	−179.1 (2)	C19—C20—C21—C22	−0.4 (5)
C15—C10—C11—N7	0.2 (4)	C18—N4—C22—C21	0.3 (4)
C9—C10—C11—C12	0.8 (4)	Ru1—N4—C22—C21	164.1 (2)
C15—C10—C11—C12	−179.9 (2)	C18—N4—C22—C23	179.1 (3)
C5—N8—C12—C13	0.7 (4)	Ru1—N4—C22—C23	−17.1 (3)
C5—N8—C12—C11	−179.1 (3)	C20—C21—C22—N4	0.2 (4)
N7—C11—C12—N8	−0.9 (4)	C20—C21—C22—C23	−178.4 (3)
C10—C11—C12—N8	179.2 (2)	N4—C22—C23—N5	31.3 (3)
N7—C11—C12—C13	179.3 (2)	C21—C22—C23—N5	−150.0 (3)
C10—C11—C12—C13	−0.6 (4)	C24—N5—C23—C22	90.0 (3)
N8—C12—C13—C14	179.4 (2)	Ru1—N5—C23—C22	−29.3 (3)
C11—C12—C13—C14	−0.7 (4)	C23—N5—C24—C25	−86.8 (3)
N8—C12—C13—C16	0.7 (4)	Ru1—N5—C24—C25	32.0 (3)
C11—C12—C13—C16	−179.5 (2)	C29—N6—C25—C26	−0.9 (4)
C8—N2—C14—C13	−0.8 (4)	Ru1—N6—C25—C26	−169.9 (2)
Ru1—N2—C14—C13	−178.5 (2)	C29—N6—C25—C24	−178.3 (3)
C8—N2—C14—C9	179.6 (2)	Ru1—N6—C25—C24	12.7 (3)
Ru1—N2—C14—C9	1.9 (3)	N5—C24—C25—N6	−30.1 (4)
C16—C13—C14—N2	1.0 (4)	N5—C24—C25—C26	152.7 (3)
C12—C13—C14—N2	−177.7 (2)	N6—C25—C26—C27	0.3 (5)
C16—C13—C14—C9	−179.4 (2)	C24—C25—C26—C27	177.3 (3)
C12—C13—C14—C9	1.9 (4)	C25—C26—C27—C28	0.4 (5)
N1—C9—C14—N2	−1.7 (3)	C26—C27—C28—C29	−0.5 (6)
C10—C9—C14—N2	177.9 (2)	C25—N6—C29—C28	0.8 (5)
N1—C9—C14—C13	178.7 (2)	Ru1—N6—C29—C28	170.0 (2)
C10—C9—C14—C13	−1.7 (4)	C25—N6—C29—C30	176.9 (3)
C4—C3—C15—C10	−0.9 (4)	Ru1—N6—C29—C30	−13.9 (4)
C4—C3—C15—C2	178.9 (3)	C27—C28—C29—N6	−0.1 (5)
C9—C10—C15—C3	180.0 (2)	C27—C28—C29—C30	−175.7 (4)
C11—C10—C15—C3	0.7 (4)	C17—N3—C30—C29	90.1 (4)
C9—C10—C15—C2	0.1 (4)	Ru1—N3—C30—C29	−28.7 (4)
C11—C10—C15—C2	−179.2 (2)	N6—C29—C30—N3	28.9 (4)
C1—C2—C15—C3	179.3 (3)	C28—C29—C30—N3	−155.4 (3)

### Hydrogen-bond geometry (Å, º)

**Table:**

*D*—H···*A*	*D*—H	H···*A*	*D*···*A*	*D*—H···*A*
N3—H3*N*···F2*A*_*a*^i^	0.91	2.22	3.037 (4)	150
N3—H3*N*···F4*A*_*a*^i^	0.91	2.55	3.236 (4)	133
N5—H5*N*···N7^ii^	0.88	2.20	3.006 (3)	153
N5—H5*N*···N8^ii^	0.88	2.69	3.373 (4)	135
C1—H1···F11*A*_*a*^iii^	0.95	2.48	3.376 (5)	157
C1—H1···F11*B*_*b*^iii^	0.95	2.28	3.019 (11)	134
C3—H3···F8*A*_*a*^iv^	0.95	2.51	3.301 (8)	141
C3—H3···F12*A*_*a*^iv^	0.95	2.60	3.414 (5)	144
C3—H3···F8*B*_*b*^iv^	0.95	2.50	3.28 (3)	140
C3—H3···F12*B*_*b*^iv^	0.95	2.37	3.283 (10)	161
C5—H5···F7*A*_*a*^v^	0.95	2.50	3.404 (5)	159
C8—H8···F1*A*_*a*	0.95	2.53	3.211 (4)	128
C8—H8···F4*A*_*a*	0.95	2.40	3.085 (4)	129
C8—H8···F1*B*_*b*	0.95	2.50	3.42 (2)	163
C17—H17*A*···N9_*a*^vi^	0.99	2.65	3.500 (8)	145
C17—H17*B*···F4*B*_*b*	0.99	2.34	3.15 (2)	138
C19—H19···F9*B*_*b*^vi^	0.95	2.61	3.287 (12)	128
C21—H21···F11*A*_*a*^vii^	0.95	2.48	3.166 (4)	129
C23—H23*B*···F6*A*_*a*^iii^	0.99	2.51	3.409 (4)	151
C24—H24*A*···F7*A*_*a*^iii^	0.99	2.53	3.224 (5)	127
C24—H24*B*···F2*B*_*b*^iii^	0.99	2.09	3.00 (2)	152
C24—H24*B*···F5*B*_*b*^iii^	0.99	2.51	3.41 (3)	150
C26—H26···F1*A*_*a*^iii^	0.95	2.55	3.328 (5)	140
C26—H26···F4*B*_*b*^iii^	0.95	2.36	3.26 (3)	156
C28—H28···N10^viii^	0.95	2.23	3.169 (12)	169
C30—H30*A*···N9_*a*^vi^	0.99	2.59	3.484 (7)	151
C30—H30*B*···F4*A*_*a*^i^	0.99	2.54	3.182 (5)	122
C32_*a*—H32*A*_*a*···F12*A*_*a*^vi^	0.98	2.36	3.273 (8)	155
C32_*a*—H32*B*_*a*···F7*A*_*a*	0.98	2.54	3.150 (7)	121
C32_*a*—H32*C*_*a*···F1*A*_*a*	0.98	2.58	3.446 (8)	148

Symmetry codes: (i) *x*+1/2, −*y*+1/2, *z*+1/2; (ii) −*x*+1, −*y*, −*z*+1; (iii) *x*−1/2, −*y*+1/2, *z*+1/2; (iv) −*x*+3/2, *y*−1/2, −*z*+3/2; (v) −*x*+3/2, *y*−1/2, −*z*+1/2; (vi) −*x*+1, −*y*+1, −*z*+1; (vii) *x*−1, *y*, *z*; (viii) *x*, *y*, *z*+1.
